# 

**Published:** 1897-04

**Authors:** 


### CONTENTS—MARCH, 1897.—VOL. XII., NO. 10.

ORIGINAL CONTRIBUTIONS—

Visceral Neurosis (Neuralgia). Byron Robinson, B. S., M. D., Chicago . . 533
Chemismus of the Stomach. Frank B. King, M. D., Houston. . . . . . . 538
On the Use of Electrolysis in Stenosis of the Cervix Uteri. A. S. Wolff, M. D., Brownsville, Texas . . . . . . . . . . . . . . . . . . . . 540
State Care of Idiots. F. R. Martin, M. D., Kyle, Texas . . . . . . . . . 547

CORRESPONDENCE—

The Proper Treatment of Abortion: Reply to Dr. Smith's Criticism. W. J. Mathews, M. D. . . . . . . . . . . . . . . . . . . . . . . . . 550

SOCIETY NOTES—

Meeting of the Missouri State Medical Association . . . . . . . . . . . . 557
Texas State Medical Association—Preliminary Announcement and Programme . . . . . . . . . . . . . . . . . . . . . . . . . . . 558

ABSTRACTS AND SELECTIONS—

Supplied Blood in Extremis—A Killer of Septicæmia and Syphilitic Virus . 563
Euhanasia . . . . . . . . . . . . . . . . . . . . . . . . . . . 565

EDITORIAL—

A New Treatment for Consumption . . . . . . . . . . . . . . . . . . 571
Legislative: The Undertaker's Bill, and Other Bills . . . . . . . . . . . 575
The "Texas Health College" Matter . . . . . . . . . . . . . . . . 577

MEDICAL NEWS AND MISCELLANY . . . . . . . . . . . . . . . . . . 579

BOOK NOTICES . . . . . . . . . . . . . . . . . . . . . . . . 582-586

PUBLISHERS' NOTES . . . . . . . . . . . . . . . . . . . . . . 586-592

